# The Association of Sorghum Growth and Physiology with Soil Carbon Sink Source Captivity in Saline Soil

**DOI:** 10.3390/plants14050670

**Published:** 2025-02-21

**Authors:** Hao Wu, Irshad Ahmad, Jiao Liu, Qianqian Zhang, Han Fei, Weicheng Bu, Guanglong Zhu, Guisheng Zhou

**Affiliations:** 1 Joint International Research Laboratory of Agriculture and Agri-Product Safety, The Ministry of Education of China, Yangzhou University, Yangzhou 225009, China; w13964483386@163.com (H.W.); irshadgadoon737@yahoo.com (I.A.); jiaoliu0407@163.com (J.L.); zqq1185151344@163.com (Q.Z.); mz120231410@stu.yzu.edu.cn (H.F.); mz120231391@stu.yzu.edu.cn (W.B.); zhuguang2007@163.com (G.Z.); 2 Jiangsu Provincial Key Lab of Crop Genetics and Physiology, Yangzhou University, Yangzhou 225009, China; 3 College for Overseas Education, Yangzhou University, Yangzhou 225000, China

**Keywords:** saline-alkali land, Jinliang 211, carbon based organic fertilizer, soil conditioners, soil source-sink characterization

## Abstract

The vast expanse of saline-alkali land in China represents a significant reserve of land resources for agricultural development. Therefore, it is essential to explore the saline-alkali tolerance of crops, the benefits of saline-alkali soil improvement, and their carbon sequestration potential. This study utilized the sorghum variety Jinliang 211 as the experimental material, conducting trials in saline-alkali woodland. A control and four different treatments combining organic fertilizers and soil amendments were established to investigate the effects of these mixtures on sorghum growth, antioxidant enzyme activity, soil improvement, and carbon sequestration characteristics. The results indicated that the combined application of organic fertilizer and rice husk biochar could enhance the salt tolerance of Jinliang 211, improve soil quality, and increase soil carbon sequestration capacity. Among the measured plant indicators, the T2 treatment (combined application of organic fertilizer and rice husk biochar) resulted in the highest dry matter accumulation, which was 68.4% higher than the control. Concurrently, the activities of antioxidant enzymes such as SOD, POD, and CAT significantly increased during the jointing stage post-treatment, with the highest enzyme activities observed in the T2 treatment. Regarding soil indicators, the soil organic carbon content initially increased and then decreased, with the T2 treatment showing the highest soil organic carbon content, 9.8% higher than the control. The soil pH initially decreased and then increased, with the T2 treatment exhibiting the lowest soil pH, 5.6% lower than the control. Importantly, the T2 treatment demonstrated the most pronounced “net carbon sink” characteristics in the soil. In summary, the T2 mixed treatment performed the best in enhancing sorghum antioxidant enzyme activity, increasing dry matter accumulation, and strengthening the carbon sequestration characteristics of saline-alkali soil.

## 1. Introduction

Salinity is a critical environmental challenge affecting crop growth and yield worldwide [1]. According to statistics, the global salinized land is about 932 million hm^2^, accounting for 20% of the arable land area. Higher salt concentration in saline soil impose osmotic stress, reducing plant ability to absorb water and as a results caused cellular hydration. Salinity leads the accumulation of soluble salts in soil or water, which creates ion toxicity, disturb nutrient uptake, and alters the nutritional imbalance (1). The excessive production of ions such as sodium (Na^+^), chloride (Cl^−^), calcium (Ca^2+^), and magnesium (Mg^2+^) etc. disrupt plant cells and involve in antioxidant activity and protein synthesis. The initial stages of crop development, especially germination and seedling emergence, are more affected by salt stress, leading to reduced germination rate, delayed seedling emergence, shorter seedling length, and reduce plant fresh and dry weights [2]. Salinity inhibits photosynthesis by reducing plant chlorophyll and limiting carbon fixation, resulting in reduced plant growth and yield [3]. The reductions in plant growth and yield due to salinity are particularly higher in arid and semi-arid areas, where extreme evaporation triggers higher salt production [4]. Therefore, to highlight the issue of salinity for sustainable agriculture productivity is very important to investigate for future food demands. Regarding this, the selection and breeding of salt-tolerant crops and the construction of salt-tolerant high-yield technology are the prerequisites for the development and utilization of saline and alkaline land for future agricultural production. In addition, determining plant antioxidant enzymes such as CAT, POD, SOD and MDA provides a comprehensive of sorghum physiological conditions under salt stress. These antioxidant enzymes demonstrated how oxidative stress management effects plant yield and soil capacity to capture and retain carbon. The measuring of plant antioxidant enzymes provides valuable insight into improving crop yield and strengthening carbon sequestration under saline soil.

Sorghum (*Sorghum bicolor* L.) is an annual C4 crop that is mainly cultivated for grain, animal feeds and fuel purposes [5,6]. Previous studies have demonstrated that sorghum showed an indispensable resistance to different abiotic stresses such as drought, flood, and nutrient imbalance [7]. Sorghum can grow normally and maintain high fresh grass yield in coastal saline soils with approximately 0.32% soil salinity [8]. However, the growth and development of sorghum can be seriously affected under higher salinity levels. Current studies on sorghum salt stress response focuses on tolerance levels, screening salt tolerance varieties, physiological and biochemical changes, hormones and nutrient regulations [9]. In this regard, Gao et al. characterized 66 sorghum germplasm varieties for salt tolerance, indicating that sorghum varieties from the Sudan grass group were more tolerant to salt, while sorghum material from the others group was more sensitive to salt [10]. Furthermore, he identified that different salt concentrations hindered seed germination, reduced seedling emergence and slowed down the growth of sweet sorghum. Salt stress can destroy the cell membrane structure, decrease the photosynthetic rate, and disturb ionic balance due to oxidative damage and osmotic stress in different crops [11]. Previous studies demonstrated that the application of N and the supply of exogenous hormones can significantly mitigate the adverse effects of salt stress [12]. However, most of the above studies were carried out indoors in hydroponic controlled trials in greenhouses, while fewer studies have been carried out under saline in situ conditions of saline soils to improve sorghum growth and yield.

Moreover, salt stress directly impacts the soil carbon sink capacity by reducing the input of organic matter from plants such root exudates, and crop residues due to osmotic stress, ion toxicity and nutrient imbalance that hinder water uptake, photosynthesis, and biomass production [13]. Salinity-induced plant stress lead to produce lower organic matter, reduced microbial activity and as a result soil lower ability to retain carbon [14]. The application of organic fertilizer to saline and alkaline land can gradually decrease total soil salinity and pH, significantly change soil indicators such as exchangeable sodium, improve soil physicochemical properties, and reduced alkali in the surface soil [15]. Previous studies demonstrated that salt stress causes plants to allocate carbon towards osmotic adjustment processes, such as osmoprotectant synthesis, rather than structural development or root exudation and as a result the overall carbon input into the soil reduces, inhibiting the soil capacity to act as carbon sink [16,17]. Soil carbon sequestration, a key component for climate change protection, relies on organic carbon deposition and stability [18]. However, in saline soils, this process is restricted by a shortage of microbial diversity and activity, which are required for organic matter decomposition and carbon stabilization. Salinity also disrupts soil organic carbon retention by disturbing soil conditions [19]. Despite these obstacles, salt-tolerant crops have demonstrated the ability to improve soil carbon dynamics in saline areas. Cinder carbon (a soil conditioner) not only reduces the degree of soil salinization, but also improves soil fertility, enhances the water-holding and water-retaining properties of the soil, and improves the plant growth environment [20]. Previous studies demonstrated that saline-tolerant crops like sorghum contribute to organic inputs to soil under adverse conditions [21]. However, the relationship of sorghum growth and physiology with soil carbon sink capacity under various organic fertilization combinations and saline soils are still unknown. Most of the previous research has primarily focused on the individual applications of these treatments on sorghum, while studies on their effects on other growth attributes remain limited.

Therefore, the main purpose of this study is performed to: (a) investigate the effects of combine of organic fertilizer, cinder carbon, rice husk biochar and microbial fertilizer on growth and physiological traits of sorghum under salinity conditions; (b) evaluate the improvement effects of different treatments on sorghum growth and their relationship with the capacity of soil carbon emission and sink; and (c) screen the best effect of combination treatment on sorghum production under saline soil conditions.

## 2. Results

### 2.1. Effect of Organic Fertilizers and Soil Amendments in Combination on Agronomic Traits of Plants

#### 2.1.1. Plant Height

**Figure 1:**
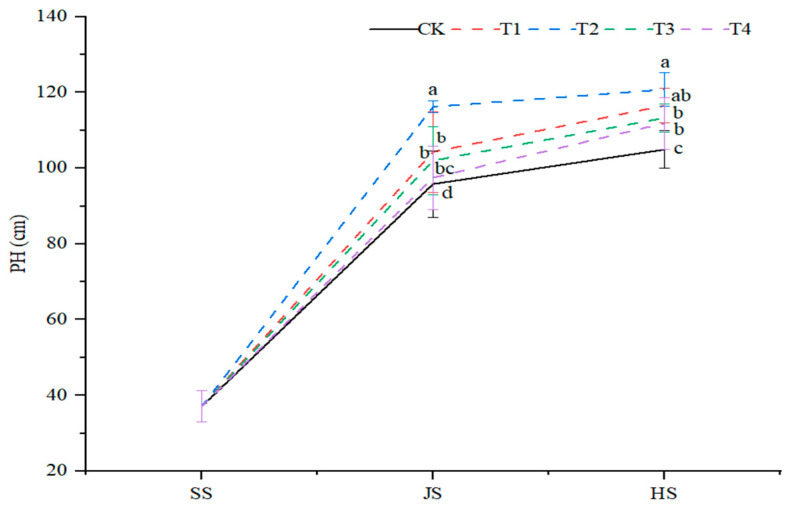
Effects of organic fertilizer combined with soil amendment on plant height of sorghum in saline soil. T1–T4 were treated with organic fertilizer and cinder carbon, rice husk biochar, bacterial fertilizer and the combination of the three-soil amendment. SS, JS and HS represent seedling, jointing and heading stages, respectively. The LSD method was used for analysis, and the letters of the same period indicated the difference at the 0.05 probability level.

Generally, sorghum plants grew faster from seedling to nodulation and slower than from nodulation to tasseling. Organic fertilizer paired with different soil amendments significantly affected sorghum plant height under all treatments, as compared with control (*p* < 0.05, Figure 1). The highest plant height was observed in the T2 treatment, followed by the T1, T3, and T4 treatments. As compared with the control, the plant height was increased by 21.3%, 8.8%, 6.4%, and 1.7% at the nodulation and by 15.1%, 11.1%, 7.9%, and 6.6% at the tasseling stage in all the four treatments.

#### 2.1.2. Biomass

The dry weight of sorghum was significantly (*p* < 0.05) affected by the grouped treatments of organic fertilizers and soil conditioners in saline soils (Table 1). At nodulation and tasseling stages, the dry weight biomass was significantly increased by 11.4%, 13.3%, 4.4%, and 47.8%, 49.8%, and 17.3% under the T1, T2, and T3 treatments, respectively, as compared with control. In addition, the biomass yield under the T4 treatment was significantly lower by 51.8% and 33.3%, as compared to the control, respectively. Whereas, at the maturity stage, the biomass of sorghum under the T1–T4 treatments were significantly higher by 12.6%, 68.4%, 12.4%, and 3.7%, as compared to the control. In addition, the sorghum biomass under the T2 treatment reached its maximum value during all growth periods.

### 2.2. Effects of Organic Fertilizers and Soil Conditioners on Membrane Lipid Peroxidation Products and Antioxidant Enzyme Activities in Plants

#### 2.2.1. MDA Content

**Figure 2:**
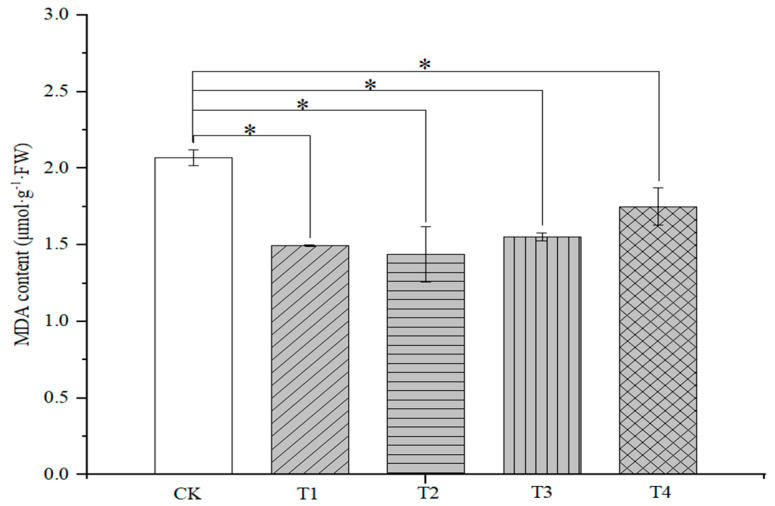
Effects of organic fertilizer combinate with soil amendment on MDA content in leaves of sorghum at jointing stage in saline soil. The T1–T4 treatments were treated with organic fertilizer and cinder carbon, rice husk biochar, bacterial fertilizer and the combination of the three-soil amendment. Analysis of variance was performed using the LSD method at the 0.05 probability, and * indicated a significant difference at the 0.05 probability level.

Sorghum leaf malondialdehyde (MDA) content was significantly (*p* < 0.05) affected by organic fertilizers paired with soil conditioners. The MDA content of sorghum leaf at 71 DAS was significantly reduced by 27.7%, 30.4%, 25.0%, and 15.5%, respectively, in all treatments, as compared with control. The greatest reduction in malondialdehyde content was observed in the T2 treatment (Figure 2).

#### 2.2.2. SOD Activity

**Figure 3:**
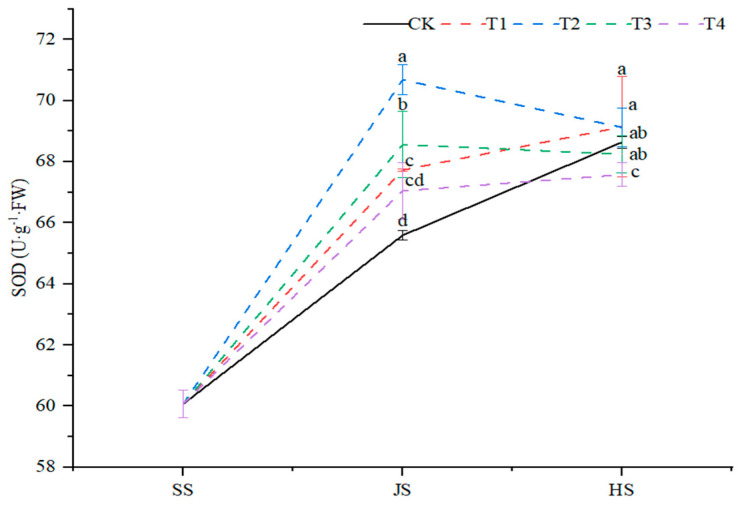
Effects of organic fertilizer combinate with soil amendment on SOD activity in leaves of sorghum in saline soil. The T1–T4 treatments were treated with organic fertilizer and cinder carbon, rice husk biochar, bacterial fertilizer and the combination of the three-soil amendment. SS, JS and HS represent seedling, jointing and heading stages, respectively. The LSD method was used for analysis, and the letters of the same period indicated the difference at the 0.05 level.

The organic fertilizer and soil conditioner combined treatments significantly (*p* < 0.05) affected SOD activity of sorghum (Figure 3). Overall, from seedling to tasseling stage, the superoxide dismutase (SOD) activity showed an increased trend and then decreased under the T2 and T3 treatments, and again a gradual increase was observed under CK in the T1 and T4 treatments. On the 71st DAS, the SOD activity was higher by 3.3%, 7.8%, 4.5%, and 2.2%, as compared to the control. The highest SOD activity was found in the T2 treatment. At the tasseling stage, the SOD activity under the T1 and T2 treatments increased by 0.71% and 0.70%, respectively, as compared with the control, but significantly decreased by 0.57% and 1.5% under the T3 and T4 treatments, respectively, as compared with the control (Figure 3).

#### 2.2.3. POD Activity

**Figure 4:**
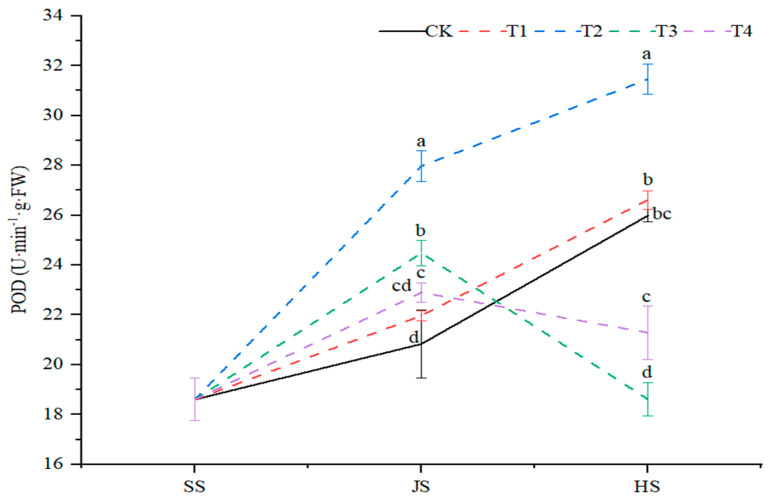
Effects of organic fertilizer combined with soil amendment on POD activity in leaves of sorghum in saline soil. T1–T4 were treated with organic fertilizer and cinder carbon, rice husk biochar, bacterial fertilizer and the combination of the three-soil amendment. SS, JS and HS represent seedling, jointing and heading stages, respectively. The LSD method was used for analysis, and the letters of the same period indicated the difference at the 0.05 level.

Organic fertilizer with soil conditioner significantly (*p* < 0.05) affected sorghum leaf peroxidase (POD) activity. The leaf POD activities of the T1 and T2 treatments and the control showed a continuous increase throughout the sorghum reproductive period, while the T3 and T4 treatments showed an increased and then decreased (Figure 4). At the nodulation stage, the POD activity under all treatments was significantly higher, with an increase of 5.5%, 34.3%, 17.5% and 9.9% as compared with the control. At the tasseling stage, the leaf POD activity was significantly increased by 21.1% and 2.4%, respectively, under the T2 and T1 treatments, as compared with the control. In addition, the POD activity was significantly decreased by 28.4% and 18.1% under the T3 and T4 treatments, as compared with control. Interestingly, the highest POD activity was recorded under the T2 treatment throughout the reproductive period.

#### 2.2.4. CAT Activity

**Figure 5:**
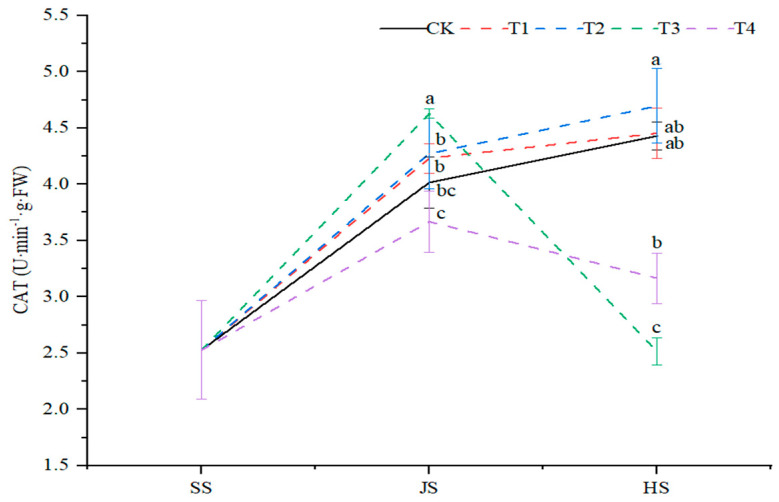
Effects of organic fertilizer combinate with soil amendment on CAT activity in leaves of sorghum in saline soil. T1–T4 were treated with organic fertilizer and cinder carbon, rice husk biochar, bacterial fertilizer and the combination of the three-soil amendment. SS, JS and HS represent seedling, jointing, and heading stages, respectively. The LSD method was used for analysis, and the letters of the same period indicated the difference at the 0.05 probability level.

Organic fertilizer paired with soil conditioner significantly (*p* < 0.05) affected sorghum leaf catalase (CAT) activity. The leaf CAT activity under CK, T1, and T2 treatments showed a continuous increase trend throughout the sorghum reproductive period. While, under T3 and T4 treatments, it showed a decreased trend after linear growth (Figure 5). At the nodulation stage, under T1, T2, and T3 treatments, the leaf CAT activity was significantly increased by 5.4%, 6.4% and 15.2%, respectively, as compared to the control. In addition, the CAT activity under T4 treatment decreased by 8.6%, as compared to the control. At the tasseling stage, the CAT activity under T2 and T1 treatments was significantly higher followed by 6.0% and 0.54%, as compared with the control. Under T3 and T4 treatments, the CAT activity was significantly decreased by 43.2% and 28.5% and was increased under T2 treatment by (4.7), as compared with the control (Figure 5).

### 2.3. Effects of Organic Fertilizer and Soil Amendments on the Characteristics of Soil Carbon Sinks and the Benefits of Amelioration

#### 2.3.1. Source-Sink Characterization of Saline Soils

At both the jointing stage and the heading stage, the co-application of organic fertilizer and soil conditioners significantly affected soil carbon emission, soil carbon sequestration, and plant carbon sequestration (*p* < 0.05) (Table 2 and Table 3).

Regarding soil carbon emission characteristics, at the jointing stage, T1, T2, and T3 treatments showed carbon sequestration. As compared with T2 and T3, T1 showed the highest carbon sequestration followed by 2.6 times and 75.7, while T4 treatment showed carbon emission. But at the heading stage, all treatments showed carbon sequestration. The T4 treatment showed the largest carbon sequestration followed by 99.4%, 99.7%, and 99.5% as compared with T1, T2, and T3 treatments, respectively. In terms of soil carbon sequestration, at the jointing stage, T2 showed the highest carbon absorption followed by 1.8 times, 2.8 times, and 2.4 times, as compared to the T1, T3, and T4 treatments. And at the heading stage, T3 treatment showed the highest soil carbon sequestration followed by 2.8 times, 1.3 times, and 2.9, as compared to the T1, T2, and T4 treatments, respectively. Regarding plant carbon sequestration, at the jointing stage, T4 resulted in the highest plant carbon sequestration followed by 2.7 times, 1.3 times, and 1.2 times, as compared to the T1, T2, and T3 treatments, respectively. But at the heading stage, T1 and T2 treatments both showed the higher carbon absorption; however, T2 showed the best performed and increased by 6.2 times than that of T1.

The net carbon emission at both the jointing stage and the heading stage was greatly influenced by plant carbon sequestration, At the jointing stage, the net carbon emissions of all treatments was less than 0, indicating a “net carbon sink” characteristic in saline-alkali soils. T4 showed the most significant carbon sink characteristic and increased carbon absorption by 173.4%, 33.8%, and 23.3%, as compared to T1, T2, and T3 treatments, respectively (Table 2). In contrast, at the heading stage, T3 and T4 treatments had net carbon emissions greater than 0, indicating a “net carbon source” characteristic in saline-alkali soils. T1 and T2 treatments had net carbon emissions less than 0, indicating a “net carbon sink” characteristic in saline-alkali soils. Compared with T1 treatments, T2 treatments showed a significant carbon sink characteristic and increased carbon absorption by 523.6 (Table 3).

#### 2.3.2. Characterization of Soil Organic Carbon in Saline Soils

**Figure 6:**
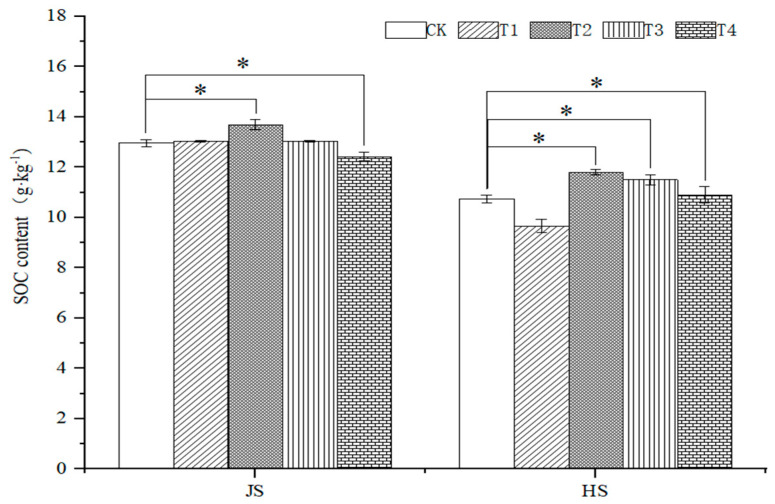
Effects of organic fertilizer combinate with soil amendment on soil organic carbon content in saline soil. The T1–T4 treatments were treated with organic fertilizer and cinder carbon, rice husk biochar, bacterial fertilizer and the combination of the three-soil amendment. JS and HS represent jointing and heading stages, respectively. Analysis of variance was performed using the LSD method, and * indicated a significant difference at the 0.05 probability level.

At the nodulation stage, the soil organic carbon under T1, T2 and T3 treatment increased by 0.62%, 7.72% and 0.54%, respectively, as compared with the control. In addition, SOC significantly decreased by 4.14%, under the T4 treatment, as compared with the control. At the tasseling stage, SOC treatment significantly decreased by 10.04% under T1, as compared with control. Moreover, the soil organic carbon under T2, T3, and T4 treatments increased by 9.77%, 6.98%, and 1.49%, respectively, as compared to the control (Figure 6).

#### 2.3.3. Characterization of pH Change in Saline Soil

**Figure 7:**
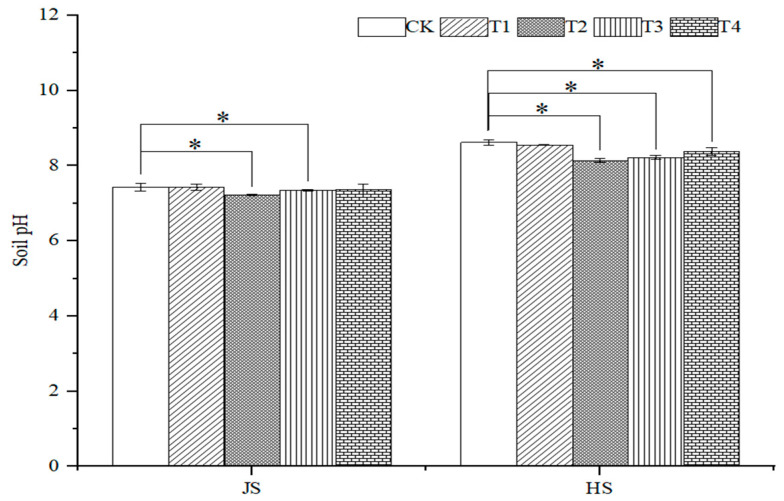
Effects of organic fertilizer combinate with soil amendment on pH in saline soil. T1–T4 were treated with organic fertilizer and cinder carbon, rice husk biochar, bacterial fertilizer and the combination of the three-soil amendment. JS and HS represent jointing and heading stages, respectively. Analysis of variance was performed using the LSD method, and * indicated a significant difference at the 0.05 probability level.

**Figure 8:**
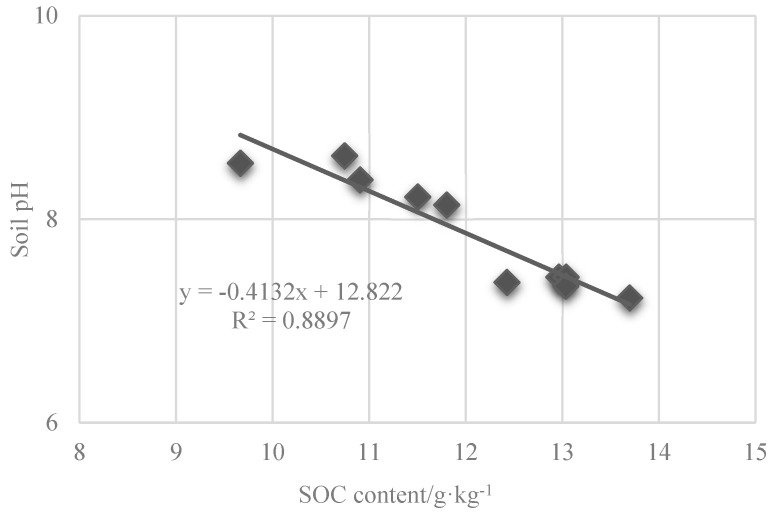
Correlation analysis between soil pH and soil organic carbon content under the condition of combined application of organic fertilizer and soil amendment. SOC represents soil organic carbon.

At the nodulation stage, the soil pH under the T1 treatment was the same as that of the control, and the soil pH under the rest of the treatments decreased, as compared with the control. The pH significantly decreased by 2.7%, 1.2% (*p* < 0.05), and 0.8% under the T2, T3, and T4 treatments as compared with control, respectively. In addition, at the tasseling stage, the soil pH under T1, T2, T3, and T4 decreased by 0.8%, 5.6%, 4.6%, and 2.7%, as compared to the control. While at nodulation stage, the soil pH increased by 15.1%, 12.6%, 11.9%, and 13.8%, as compared with control (Figure 7 and Figure 8).

## 3. Discussion

Soil salinization poses a significant challenge to agricultural productivity and land-use efficiency [22]. This study explores the synergistic effects of combining organic fertilizers with soil conditioners on the salt tolerance of the Jinliang 211 sorghum variety and the amelioration of saline soils. The findings reveal that the T2 treatment, which involved a mixture of organic fertilizer and rice husk biochar, significantly enhanced the salt tolerance of Jinliang 211 and improved the condition of saline soils under equivalent nitrogen application levels. Sodium (Na^+^) and chloride (Cl^−^) ions, prominent in saline soils, induce oxidative damage, osmotic stress, ionic toxicity, and growth inhibition in plants [23,24].

At the nodulation stage of Jinliang 211, the T2 treatment resulted in lower malondialdehyde (MDA) content compared to the control (CK) and other treatments (T1, T3, T4), indicating reduced oxidative stress. Concurrently, the activities of superoxide dismutase (SOD) and peroxidase (POD) were elevated across all treatments relative to CK, with T2 exhibiting the highest enzyme activities. These observations suggest that saline conditions initially impose osmotic stress and subsequently trigger reactive oxygen species (ROS) production in plant cells [25]. Supporting these findings, Saha and Singh et al. documented increased MDA levels and membrane peroxidation under saline stress [26,27]. The application of organic fertilizer combined with rice husk biochar effectively mitigated osmotic stress and enhanced antioxidant enzyme activities, thereby bolstering the salt tolerance of Jinliang 211. This aligns with Chutipaijit et al. assertion that rice varieties exhibiting lower MDA production under salt stress demonstrate greater resilience to cellular damage [28]. Similarly, Kamaei and Reza et al. noted that organic fertilizers can enhance plant growth by reducing Na^+^ and Cl^−^ concentrations [29].

The study further observed varying trends in SOD, POD, and catalase (CAT) activities across different growth stages of Jinliang 211, from nodulation to tasseling. The T2 treatment consistently showed the highest enzyme activities at the tasseling stage, whereas T3 and T4 treatments underperformed relative to CK, suggesting a diminished salt tolerance in later growth stages. This was reflected in the plant height and dry matter weight of Jinliang 211, which were only marginally higher in T3 and T4 treatments compared to CK at the tasseling stage.

In terms of soil improvement, enhancing the nutrient efficacy of saline soils is crucial. Soil organic matter, a vital nutrient source, facilitates soil structure formation and improves physicochemical properties [30]. Soil organic carbon, a key component of soil organic matter, plays a pivotal role in soil fertility and the global carbon cycle [31]. The T2 treatment reduced soil pH by 5.6% and increased soil organic carbon content by 7.7% and 9.8% at the nodulation and tasseling stages, respectively, thereby enhancing soil fertility. These results corroborate Xiao et al. findings, which demonstrated that bio-organic fertilizers combined with soil conditioners significantly lower the pH of coastal saline soils [32].

Regarding soil carbon dynamics, the T1 and T2 treatments exhibited “net carbon sink” characteristics during the nodulation and tasseling stages of Jinliang 211, with T2 showing the most pronounced carbon sequestration. Conversely, T3 and T4 treatments did not exhibit this feature. Additionally, the study noted an increase in plant carbon sequestration and a significant reduction in net soil carbon emissions. Hu et al. similarly observed that exogenous organic carbon application enhances soil carbon sequestration and reduces net carbon emissions [33]. The current study’s findings align with Zhang et al.’s research, which also reported a decrease in net carbon emissions with increased plant carbon sequestration following the application of organic fertilizers and soil conditioners [34].

In conclusion, the combined application of organic fertilizer and rice husk biochar (T2) not only improves the salt tolerance of Jinliang 211 but also enhances soil quality and carbon sequestration, offering a sustainable approach to managing saline soils.

In this study, we selected Jinliang 211 sorghum variety and treated it with the combine application of organic fertilizer and soil conditioner. A fertilizer application method (3000 kg ha^−1^ organic fertilizer mixed with 600 kg ha^−1^ rice husk biochar) was provided for growing sorghum in saline soil. Most of the previous studies have focused on the effects of organic fertilizers or soil conditioners alone on crops and soils [35]. However, in this study, the two fertilizer treatments were applied together to investigate the effects of salt stress on sorghum under saline soil conditions. On the other hand, this study correlated the aboveground biomass of sorghum plants with the net soil carbon emissions, and analyzed the carbon sink characteristics of the soil using the carbon sequestration capacity of sorghum plants. Moreover, previous studies were conducted in greenhouse gas emission fluxes to characterize the soil sinks capacity, and the interactions between the soil and the ground part of the plant [36].

In the current study, the observed enhancement in the flexibility of Jinliang 211 sorghum is likely attributed to the combined application of organic fertilizer and rice husk biochar under saline soil conditions. However, to fully understand the underlying mechanisms, further research is necessary to investigate the genetic background of the Jinliang 211 variety and its specific responses to saline stress. Additionally, more studies are needed to elucidate the interrelationships between changes in the physiological characteristics of the plant, the duration of action of organic fertilizers mixed with rice husk biochar, and the varying degrees of soil salinity during the later growth stages of Jinliang 211. Such investigations would help clarify the mechanisms of salt tolerance in sorghum plants and optimize the application of these amendments for improved crop performance.

This study concluded that Jinliang 211 exhibited the highest carbon sequestration capacity and soil carbon sink potential when treated with a combination of organic fertilizer and rice husk biochar. Nevertheless, to validate and expand upon these findings, further field studies involving a broader range of sorghum varieties are essential. These studies should aim to explore the mechanisms underlying the effects of organic fertilizer and rice husk biochar application, as well as to establish a clearer relationship between sorghum growth, yield, and soil carbon sink capacity. Such efforts would contribute to the development of sustainable agricultural practices for saline soil management and enhance the resilience of sorghum cultivation in challenging environments.

## 4. Materials and Methods

### 4.1. Experimental Site

A field experiment was conducted during two consecutive year of 2023 and 2024 at the Dafeng Coastal Forest Farm in Yancheng City, Jiangsu Province (33°20′ N, 120°47′ E). The experimental region is characterized by a climate transitioning from subtropical to warm and humid temperate zones, with distinct seasons, moderate temperatures, an average annual temperature of 14.1 °C, abundant rainfall with an annual precipitation of 1042.2 mm, a frost-free period of 213 days, and annual sunshine hours of 2238.9 h.

The sample site is a chalky sandy silt soil with organic matter content 21.43 g·kg^−1^, total N 0.81 g·kg^−1^, available P 1.51 g·kg^−1^, available K 266.00 g·kg^−1^. The soil pH was 8.1, the salt content was 2.1 g·kg^−1^, and it had an average electrical conductivity (EC) of 17.52 mS cm^−1^ in the 0–20 cm soil layer. The chalky sandy silt soil contained more than 50% silt, less than 50% sand and less than 15% minimal clay content. The soil textures of the field were fine and smooth but not sticky like a clay soil with moderate nutrients retention and high erosion susceptibility.

The soil information of the current experimental plots mainly belongs to the Solonchaks and Solonetz categories according to the FAO soil classification system [37]. Through prolonged natural processes and human-induced enhancements, the majority of heavily saline soils have progressively transformed into moderately and lightly saline soils, with soil salinity levels exhibiting variability across different plots.

### 4.2. Sorghum Variety and Soil Conditioners

The sorghum variety, Jinliang 211, known for its adaptability, tolerance to salinity and alkalinity, was used in the present study. The thousand kernel weight was 26.5 g. The seeds were provided by the Sorghum Institute of Shanxi Agricultural University. The seeds were well-preserved, vigorous, intact, and free from pests under laboratory conditions.

The organic fertilizer and soil conditioners (rice husk biochar and cinder carbon) were provided by Nanjing Forestry University. The organic fertilizer (NY/T525-2021) was carbon-based with equal percentages of 4% N, P_2_O_5_ and K_2_O [38]. Rice husk biochar was made from rice husk with a pyrolysis temperature of 550 °C and a residence time of 90 min. The main components were cellulose, lignin and water in the ratio of 5:3:2, with small amounts of silica (SiO_2_), alumina (Al_2_O_3_) and iron oxide (Fe_2_O_3_), and the pH value was 7.52. It had physical properties such as gray color, irregular texture, <45 nm in size and odorlessness, etc. At 550 °C, the rice husk biochar formed many pores, with an average pore size of 3.77 nm, and the volume of the micropores was 0.015 cm^3^·g^−1^. The main components of cinder carbon are silicon dioxide (SiO_2_), aluminum oxide (Al_2_O_3_) and iron oxide (Fe_2_O_3_), with SiO_2_ content of about 46–62%, and other inorganic ash such as calcium oxide (CaO), magnesium oxide (MgO) and sulfur trioxide (SO_3_), which are weakly acidic. It is characterized by low carbon content, low calorific value and hard texture. Its particle size is large, with porous, adsorption and adhesion properties. The microbial fertilizer (NY884-2012, Caile Plant Factory, Huaian, China) is a commercial product, consisting of efficient bacteria such as *Bacillus amyloliquefaciens* and *Bacillus subtilis*, with a minimum viable bacterial count of ≥300 million g^−1^.

### 4.3. Experimental Design

The experiment was designed with four combination treatments and a control, (1) organic fertilizer and cinder carbon blend (T1); (2) organic fertilizer and rice husk biochar blend (T2); (3) organic fertilizer and microbial fertilizer blend (T3); (4) Organic fertilizer mixed with cinder carbon, rice husk biochar and microbial fertilizer (T4); (5) Control without fertilizer combination treatment (CK) (Table 4).

A single factor randomized block design was used, with three replicates for each treatment, resulting in a total of 12 plots, each with an area of 20 m^2^. Sorghum seeds were sown directly by ridge planting on June 25 in both cropping years at a seeding rate of 22.5 kg ha⁻^1^. The total nitrogen application during the entire growth period was 240 kg ha⁻^1^, using compound fertilizer with equal percentages of 15% N, P_2_O_5_ and K_2_O, which were applied in a ratio of 4:3:3 as basal, seedling, and tillering fertilizers, respectively. Under the condition that the total nitrogen application was fixed for the entire growth period of sorghum in each plot, the fertilizers corresponding to each mixed treatment were applied in combination with seedling fertilizer. The basal, seedling, and tillering fertilizers were applied in strips and thoroughly incorporated into the top 0–20 cm layer of tilled soil.

### 4.4. Observations and Measurements

#### 4.4.1. Determination of Growth Characteristics and Physiological Properties of Sorghum

On the 42nd, 71st and 103rd days after sowing (DAS) (at seedling, jointing and heading stages of sorghum, respectively), nine representative plants were sampled from each plot for measurements. The plant height and leaf area were determined and then kept in an oven until constant weight for the final dry weight determination. On the 71st DAS (nodulation stage), 5 more plants were collected, and the upper fresh leaves were randomly selected and frozen in liquid nitrogen and stored in an ultra-low-temperature (−80 °C) refrigerator for the subsequent determination of antioxidant enzyme activities and osmoregulatory substance contents.

Catalase (CAT) activity: About 0.1 g of frozen leaf was homogenized in 5 mL assay mixtures which contained 2.9 mL substrate solution (30% hydrogen peroxide in 50 mmol L^−1^ potassium phosphate buffer) and 0.1 mL of enzyme extraction. The decomposition of H_2_O_2_ was stopped by adding 2 mL potassium-dichromate (5%) to the mixed solution. The absorbency was measured immediately at 240 nm and read every 30 s for 2 min to calculate the CAT enzyme activity.

Superoxide dismutase (SOD) activity: 1.0 g of frozen leaf was ground with 3 mL of 0.05 mol L^−1^ PBS buffer (pH = 7.8) and a small amount of quartz sand in an ice bath, then transferred into a 5 mL centrifuge tube, and centrifuged at 10,000 r min^−1^ for 15 min at 4 °C, and the supernatant was extracted as the enzyme solution. The SOD reaction solution (5 mL 100 mmol L^−1^ potassium phosphate buffer (pH 7.8) which containing 0.1 mmol L^−1^ EDTA (ethylenediamine tetraacetic acid disodium salt), 0.1% Triton X-100 and 2% polyvinyl pyrrolidone) and 1 mL enzyme solution were taken in 10 mL centrifuge tubes, and immediately subjected to fluorescent tube light at 4000 LX, and the reaction was terminated by stopping the light and shading the light after 15 min. The absorbency was measured at 560 nm wavelength colorimetric. One unit of SOD activity is expressed as the amount of enzyme required to cause 50% inhibition of epinephrine oxidation [38].

Peroxidase (POD) activity: About 0.1 g frozen leaf was ground in 3 mL of 0.1 mol L^−1^ phosphate buffer (pH 7.0) to extract POD. After that, the extraction was centrifuged at 18,000× *g* at 4 °C for 15 min. The supernatant was used as the enzyme source. The oxi-dized o-diphenylamine was determined at 430 nm. Phosphate buffer (0.1 mol L^−1^, pH 6.5) was placed in colorimetric dishes containing enzyme extract. Then, 0.2 mL 0.2 mol L^−1^ H_2_O_2_ was added and mixed, and the absorbance per min was recorded. The POD activity unit expressed as the rate of increase in absorbance per min [39].

Malonaldehyde (MDA) content: About 0.5 g of fresh leaf was ground in 0.1% tri-chloroacetic acid (TCA), then mixed and centrifuged at 12,000× *g* for 15 min to prepare for the MDA extraction. After that, 1 mL supernatant with 4 mL 0.5% thiobarbituric acid (containing 20% trichloroacetic acid) was heated at 95 °C for 15 min and then centrifuged at 10,000× *g* for 15 min. Then, the sample was recorded for the absorption at 600, 532, and 450 nm and MDA content were calculated [40].

#### 4.4.2. Determination of Soil Indicators

For each plot, surface soil samples from a depth of 0–20 cm were collected using the five-point sampling method, stored protected from light and naturally air-dried.

Soil pH: After air-drying the soil, impurities were removed, and the soil was ground and sieved (2 mm mesh). According to a soil-to-water volume ratio of 2.5:1, 10 g of soil sample was weighed and placed into a small beaker. Then, 25 mL of CO_2_-free distilled water was added, the mixture was stirred thoroughly for 5 min, and left to stand for 30 min. The soil pH was measured using a pH meter at room temperature (25 °C).

Electrical Conductivity (EC): According to a soil-to-water mass ratio of 5:1, 20 g of air-dried soil sample (passed through a 2 mm sieve) was weighed and poured into a conical flask. Then, 100 mL of distilled water was added, and the mixture was shaken vigorously on a shaker for 3 min. The mixture was then transferred to a centrifuge tube and centrifuged at 4000 rpm for 30 min. The supernatant was further filtered, and 20 mL of the filtrate was taken to measure the electrical conductivity using a conductivity meter at room temperature (25 °C).

Salt Content (Mass Fraction of Water-Soluble Salts): A clean and dry glass test tube was precisely weighed using an electronic balance (accurate to 0.1 mg). Then, 50 mL of the above filtrate was accurately measured and placed into the test tube. The filtrate was evaporated to dryness in a water bath, and the residue was dried in an oven set at 105 °C until a constant mass was achieved. The mass was precisely weighed, and the soil salt content was calculated [41].

#### 4.4.3. Measurement of Soil Carbon Emission Characteristics

Gas collection: Use a static box, which consists of two parts: the base and the box, which has a built-in fan for mixing the gas in the box. The gas was sampled at the nodulation and tasseling stages of the treated sorghum plants, respectively, with no sorghum plants in the box. The sampling interval was 10 min, 0, 10, 20 and 30 min after the box was placed in each test plot, respectively, and the gas was collected in a single-valve aluminum foil gas sampling bag with a volume of 100 mL by connecting the box through a gas sampler for measurement.

Gas analysis: The gas samples were measured by gas chromatograph (Agilent 7890A), the working conditions of which are shown in (Table 5). The gas samples of each batch were analyzed and measured within 72 h, and the main gases measured were methane (CH_4_) and carbon dioxide (CO_2_).

Data computation:

(1) $$CE=\sum_{i=1}^{n}(\frac{F_{i}+F_{i+1}}{2})\times (t_{i+1}-t_{i})\times 24$$

In Formula (1), CE denotes cumulative gas emissions, mg·m^−2^; F is the gas emission flux, mg·m^−2^·h^−1^; i denotes the ith gas sampling; t_i+1_ − t_i_ denotes the interval between two adjacent measurement dates, d; n is the total number of measurements during the cumulative emissions observation time.

(2) $$Rm=\left\{\frac{12}{16}\times \left[T_{i}{CE}_{{CH}_{4}}-CK{CE}_{{CH}_{4}}\right]+\frac{12}{44}\times [T_{i}{CE}_{{CO}_{2}}-CK{CE}_{{CO}_{2}}]\right\}\times \frac{16}{1000}$$

In Formula (2), Ti denotes individual treatments; CE_CH4_ denotes cumulative CH_4_ emissions, g·m^−2^; CE_CO2_ denotes cumulative CO_2_ emissions, g·m^−2^.

(3) $$SCS=\left[T_{i}SOC\times T_{i}\rho b-CKSOC\times CK\rho b\right]\times h\times S/10^{6}$$

In Formula (3), SOC denotes soil carbon content, g·kg^−1^; ρb denotes soil bulk weight, g·cm^−3^; S is the unit plot area, cm^2^; h is the height of the soil layer, cm.

(4) $$NPP=\left(T_{i}FW-CKFW\right)\times \left(1-WS\right)\times n\times 0.45$$

In Formula (4), FW denotes plant fresh weight, kg; WS denotes plant water content; n is the number of plants; 0.45 is the conversion factor of plant biomass to carbon content.

(5) $$NCE=Rm-\left(SCS+NPP\right)$$

### 4.5. Statistical Analysis

The experimental data were collated and plotted using Origin 2024, and the data were statistically analyzed using Statistix 9.0 (Analytical Software, Tallahassee, FL, USA). The mean values were compared based on the least significant difference (LSD) test at *p* < 0.05. All the parameters are shown as the average values of the two-year experiments because the tendency of each parameter was similar in each year and there was no significant difference between the two years.

## 5. Conclusions

Compared to the control group without organic fertilizer and soil amendment mixtures, the T1–T4 treatments demonstrated a certain degree of enhancement in plant adaptation, soil carbon sink capacity, and soil improvement under saline cultivation conditions. Among these, the T2 mixed treatment exhibited the most significant improvement in plant salt tolerance and soil carbon sink characteristics, primarily by boosting antioxidant enzyme activities, enhancing plant carbon sequestration, and maximizing the benefits of soil amendments. Consequently, this study recommends the application of 3000 kg ha⁻^1^ organic fertilizer combined with 600 kg ha⁻^1^ rice husk biochar as an effective fertilization strategy.

## Figures and Tables

**Table 1:** Effects of organic fertilizer combined with soil amendment on biomass of sorghum in saline soil.

Crop	Treatment	Dry Weight/kg·ha^−1^		
		JS	HS	MS
Jinliang 211	CK	2025.0 c	5028.0 d	10,344.0 d
	T1	2256.0 a	7434.0 b	11,652.0 b
	T2	2295.0 a	7533.0 a	17,424.0 a
	T3	2115.0 b	5898.0 c	11,622.0 b
	T4	975.0 d	3354.0 e	10,722.0 c
SEM		1.57	5.33	3.85
CV		6.90	7.77	2.64

The T1–T4 treatments were treated with organic fertilizer and cinder carbon, rice husk biochar, bacterial fertilizer and the combination of the three-soil amendment. JS, HS and MS represent jointing, heading and maturation stages, respectively. SEM and CV represent standard error of the mean coefficient of variation, respectively. The LSD method was used for analysis, and the letters of the same period indicated the difference at the 0.05 probability level.

**Table 2:** Analysis of the source-sink characteristics of soil under the organic fertilizer combinate with soil amendment at the jointing stage of sorghum.

Treatment	Rm/kg·plot^−1^	SCS/kg·plot^−1^	NPP/kg·plot^−1^	NCE/kg·plot^−1^
CK	-	-	-	-
T1	−2.204 × 10^−9^ d	0.079 b	28.596 d	−28.676 a
T2	−8.377 × 10^−10^ c	0.142 a	58.589 c	−58.589 b
T3	−2.908 × 10^−11^ b	0.051 d	63.607 b	−63.607 c
T4	5.526 × 10^−10^ a	0.060 c	78.339 a	−78.399 d
SEM	0.11	0.0086	2.26	1.81
CV	−25.23	14.78	5.60	−4.44

Rm: soil carbon emissions; SCS: soil carbon sequestration; NPP: plant carbon sequestration; NCE: net carbon emissions. Negative values indicate carbon absorption (carbon sequestration), while positive values indicate carbon emissions. The T1–T4 treatments were treated with organic fertilizer and cinder carbon, rice husk biochar, bacterial fertilizer and the combination of the three-soil amendment. SEM and CV represent standard error of the mean coefficient of variation, respectively. The LSD method was used for analysis, and the letters of the same period indicated the difference at the 0.05 probability level.

**Table 3:** Analysis of the source-sink characteristics of soil under the organic fertilizer combinate with soil amendment at the heading stage of sorghum.

Treatment	Rm/kg·plot^−1^	SCS/kg·plot^−1^	NPP/kg·plot^−1^	NCE/kg·plot^−1^
CK	-	-	-	-
T1	−2.823 × 10^−9^	−0.051 d	15.113 b	−15.062 c
T2	−1.824 × 10^−9^	0.107 b	93.817 a	−93.924 d
T3	−2.713 × 10^−9^	0.140 a	−76.556 c	76.415 b
T4	−5.521 × 10^−7^	0.049 c	−98.856 d	98.807 a
SEM	0.15	0.01	2.42	1.46
CV	−15.23	31.83	−20.83	−8.55

Rm: soil carbon emissions; SCS: soil carbon sequestration; NPP: plant carbon sequestration; NCE: net carbon emissions. Negative values indicate carbon absorption (carbon sequestration), while positive values indicate carbon emissions. The T1–T4 treatments were treated with organic fertilizer and cinder carbon, rice husk biochar, bacterial fertilizer and the combination of the three-soil amendment. SEM and CV represent standard error of the mean coefficient of variation, respectively. The LSD method was used for analysis, and the letters of the same period indicated the difference at the 0.05 level.

**Table 4:** The mixed application method and dosage of each treatment.

Treatment	Organic Fertilizer Application Rates/kg·ha^−1^	Soil Amendment	Soil Amendment Application Rates/kg·ha^−1^
CK	-	-	-
T1	3000.0	CC	600
T2	3000.0	RHB	600
T3	3000.0	MF	600
T4	3000.0	CC + RHB + MF	200 + 200 + 200

CC, RHB and MF represent cinder carbon, rice husk biochar and microbial fertilizer, respectively.

**Table 5:** Working conditions of gas chromatograph.

Temperature/°C				Gas Flow Rate/L·min^−1^			Output Value	
Column Box	Catalyzer	Pre-Detector	Post-Detector	Exhaust	Gas	Fuel Gas	Pre-Detector	Post-Detector
30	275	230	330	2.000	40.0	400.0	16–17	220–240

## Data Availability

Data are contained within the article.
